# COVID-19 diagnostic assays sensitivity: lessons for the upcoming wave or next pandemic

**DOI:** 10.4155/fmc-2021-0209

**Published:** 2021-09-02

**Authors:** Nikhil Shri Sahahjpal, Ashis K Mondal, Sudha Ananth, Kimya Jones, Alka Chaubey, Ravindra Kolhe

**Affiliations:** ^1^Department of Pathology, Medical College of Georgia, Augusta University, Augusta, GA, USA; ^2^Bionano Genomics Inc., San Diego, CA 92121, USA

**Keywords:** assay sensitivity, COVID-19, diagnostic assays, next wave, NGS, pandemic, RT-PCR

COVID-19 diagnostic assays have played an essential role in the current pandemic and have remained at the forefront of determining critical healthcare, economic and governance policies around the globe. Several classes of COVID-19 diagnostic assays that aim to detect the SARS-CoV-2 virus in the acute phase of infection have been employed – namely, reverse-transcription PCR (RT-PCR), digital PCR (dPCR), RT loop-mediated isothermal amplification (RT-LAMP), transcription-mediated amplification (TMA), next-generation sequencing (NGS) and antigen-based detection assays [1]. Of these diagnostic assays, antigen- and RT-PCR-based assays, which are inherently different in principle, have been employed globally to cater to different populations and address evolving situations in the pandemic. Several antigen-based assays were employed in the first phase of the pandemic for rapid detection of SARS-CoV-2 as point-of-care tests worldwide. These immunological assays deploy antibodies to search for the presence of viral antigens viz. spike glycoprotein (S) or nucleocapsid protein (N) in patients' samples. The RT-PCR-based assay reverse transcribes viral RNA into cDNA, and then amplifies regions of the cDNA to levels sufficient to detect targeted conserved regions of the SARS-CoV-2 genome [2]. RT-PCR assays have been used to identify the presence of the SARS-CoV-2 in saliva [3], blood [4], rectal swabs, urine, stool [5], nasopharyngeal swabs, oropharyngeal swabs, nasopharyngeal washes, nasal aspirates, sputum, bronchoalveolar lavage (BAL) fluid and tracheal aspirates [6].

However, the sensitivity of these commercially available COVID-19 diagnostic assays varies enormously in a theoretical range of more than 10^4^. The assays in the high limit of detection (LoD) range would result in a high percentage of false-negative results compared with highly sensitive RT-PCR-based methods [7]. With highly sensitive RT-PCR assay, the reporting of high Ct value results has been under scrutiny because these results might be PCR artifacts or the clinical significance of these values is not yet completely understood [8]. Herein, we compare the clinical sensitivity of commercially available COVID-19 diagnostic assays by comparing antigen-based assays with a highly sensitive RT-PCR assay and also compare two RT-PCR-based assays. Further, we discuss the significance of high-Ct results obtained with a highly sensitive RT-PCR assay.

## Antigen-based assays compared with highly sensitive RT-PCR-based assay

A highly sensitive PerkinElmer (PE) RT-PCR assay based on nucleic acid extraction followed by TaqMan-based amplification of two target SARS-CoV-2 genomic regions (nucleocapsid [*N*] and *ORF1ab* gene) was validated for NPS and saliva samples with an LoD of 20 and 60 copies/ml, respectively (PerkinElmer New Coronavirus Nucleic Acid Detection kit, FDA-EUA assay by PerkinElmer Inc., MA, USA) [9]. Seven samples previously tested using the PE RT-PCR assay, ranging from 15 to 20, 20 to 25, 25 to 30 and 30 to 35 Ct values each, were evaluated with four antigen-based SARS-CoV-2 detection assays (targeting N1, N2, N3 and S and N) domain of SARS-CoV-2 virus. The antigen-based assays were able to detect NPS samples with Ct values up to 25 and showed false-negative results for a sample with >25 Ct values. Thus, the antigen-based assays would result in a high percentage of false-negative results, and their clinical utility should be reassessed. The antigen-based tests are useful for rapid screening, as point-of-care tests, and must be used for COVID-19 screening of symptomatic patients because rapid detection enables the ability to undertake the necessary measures, but a negative result should always be followed by a sensitive RT-PCR test. Thus, the antigen-based tests should only be used as a quick screening test and must be followed by a diagnostic assay such as RT-PCR or serology-based assay.

## Comparison of two commercially available RT-PCR assays

Two RT-PCR-based SARS-CoV-2 detection assays (Luminex and PerkinElmer Inc.) were validated as per US FDA guidelines. The Luminex assay is a one-step multiplex TaqMan-based RT-PCR assay with RNA extraction, DNA amplification and fluorescence detection occurring in a single cartridge. The assay detects N1 and N3 targets of the *N* gene, with the *RNaseP* gene serving as the housekeeping control. The Luminex RT-PCR assay was validated for NPS samples with an LoD of 1500 copies/ml. The PE RT-PCR assay was validated for NPS and saliva samples with an LoD of 20 and 60 copies/ml. Twenty samples were evaluated with both Luminex and PE RT-PCR assay. The Ct values were found to be higher with Luminex RT-PCR assay compared with PE RT-PCR assay, with samples >36 Ct not detected with Luminex assay. The data demonstrate the variable limit of detection of the two assays based on the same principle, which is RT-PCR-based amplification and detection of SARS-CoV-2 target regions. The two RT-PCR methods compared in this study vary in the sensitivity >100-fold; however, the assays available commercially vary by more than 10,000-fold. Thus, the assay with the high LoD would lead to a high percentage of false-negative results and would lead to active transmission of the SARS-CoV-2 virus and have serious public health consequences.

## Clinical significance of high Ct results obtained with a highly sensitive RT-PCR assay

We had previously sequenced 827 samples ranging from 10 to 42 Ct values, of which 95 samples were with Ct value >37 with PE RT-PCR assay [10]. Of the 827-sample sequenced, the percentage of sequencing reads that aligned to the SARS-CoV-2 Wuhan-hu-1 reference genome (NC_045512.2) ranged from 5.1 to 100%. All samples sequenced showed high sequence specificity to the SARS-CoV-2 virus. Low-Ct samples showed complete uniform coverage across the entire 29 kb SAR-CoV-2 genome, whereas the coverage in samples with high-Ct (>37) ranged from 5.1% to 99.9%, and a gradual decrease in coverage uniformity was observed with increasing Ct values [10]. The results of our study confirmed that samples with >37 Ct value reported with PE RT-PCR assay were true positive results and not PCR artifacts or contamination. There are several insights from the sequencing results of samples with high Ct values. First, it is interesting to note that the coverage in these samples ranged from 5.1 to 99.9%. Decision analytical models have assessed that 59% of all transmission are asymptomatic transmission, comprising 35% from presymptomatic and 24% from asymptomatic individuals, and thus early detection of infected individuals (high Ct results) in the presymptomatic phase of the disease using highly sensitive RT-PCR methods becomes an imperative measure that can impede the transmission of the SARS-CoV-2 virus. However, on the contrary, the reporting of high Ct results has been criticized because these results might be falsely positive or represent viral shedding and are not active virus particles that can be transmitted. Thus, although it becomes important to report the high Ct results, it is impossible to distinguish whether the infected individual is in the presymptomatic stage or is toward the late phase of infection with an RT-PCR report. However, the variable sequence coverage observed in our study hints toward a trend in which high Ct results in the presymptomatic stage might result in full uniform coverage of the SARS-CoV-2 genome, whereas those in the later phase might only result in partial coverage. To ascertain the observed trend, we need longitudinal studies to determine whether this hypothesis holds and NGS would be able to distinguish early- and late-phase infections.

In our study, we confirmed that the high Ct value results are true positives; another argument for not reporting high Ct results has been that it is not an active infection but only late-phase viral shedding. Although we have already argued that high Ct results are observed in both early and late phases of infection, and even if we can distinguish between early and late infections, it is important to report not only for early- but also late-infection results. The lack of reporting high Ct late-phase infection results would have a multifold impact on efforts to prevent transmission. Firstly, it would derail any efforts for contact tracing because the subject would be resulted negative for COVID-19. Thus, all individuals that this subject would have contacted during the active phase would not be diagnosed, and inappropriate quarantine time would lead to active transmission in the community. Further, resulting these high Ct samples as negative for COVID-19 has epidemiological consequences because they lead to incorrect infection rates in the public health database.

## Lessons learned for next wave or pandemic

In the current pandemic, we have seen massive spikes in the infection rates throughout the year, at different times in several regions of the world. We have seen the use of several assays with different LoD/sensitivity, around the globe; although this was necessary, it seems to have contributed to the spread of the virus. It would be important to make distinctions between screening and diagnostic tests; for example, the antigen-based assays (assays with high LoD) would serve as a screening test and RT-PCR-based assays (assay with low LoD) would serve as diagnostic tests. Also, there is a need for uniformity among the commercially available RT-PCR tests and an LoD cutoff for clinical approval. Regulatory authorities must determine an LoD/sensitivity cutoff above which an assay should not be approved for clinical diagnosis. NGS technology has played a significant role in the current pandemic, but massive sequencing efforts were only undertaken after the new variants were already in circulation and had led to spikes in the infectivity rate in certain regions of the world. Thus, sequencing efforts should accompany the diagnosis from the beginning to monitor phylogenetic variation and predict transmission in the community, which would also enable regulatory authorities to make recommendations and public healthcare policies.
